# Supplementary material for: Determinants of 1-Year Adverse Event Requiring Re-Hospitalization in COVID-19 Oldest Old Survivors
Source: Geriatrics (Basel). 2023 Jan 10;8(1):10. doi: 10.3390/geriatrics8010010 (PMC9844439; doi:10.3390/geriatrics8010010)
Supplement: Supplementary file 1 [file geriatrics-08-00010-s001.zip › geriatrics-2136433-supplementary.pdf]

Table S1. 30-Items Frailty Index.

FRAILTY INDEX 30 ITEMS	0 POINT	0.5 POINT	1 POINT
**Bathing**	Preserved	Impaired	Lost
**Dressing**	Preserved	Impaired	Lost
**Toileting**	Preserved	Impaired	Lost
**Transferring**	Preserved	Impaired	Lost
**Continence**	Preserved	Impaired	Lost
**Feeding**	Preserved	Impaired	Lost
**Ability to Use Telephone**	Preserved	Impaired	Lost
**Shopping**	Preserved	Impaired	Lost
**Food Preparation**	Preserved	Impaired	Lost
**Housekeeping**	Preserved	Impaired	Lost
**Laundry**	Preserved	Impaired	Lost
**Mode of Transportation**	Preserved	Impaired	Lost
**Responsability for Own Medications**	Preserved	Impaired	Lost
**Ability to Handle Finances**	Preserved	Impaired	Lost
**BMI**	< 25	25-29	≥ 30
**SPSMQ**	0-3	4-7	≥ 8
**MNA-sf**	12-14	8-11	≤ 7
**CIRS**	0	1-2	≥ 3
**Number of Medication**	0-3	4-6	≥ 7
**Co-habitation Status**	Living with Family	Institutionalized	Living Alone
**SPPB**	10-12	7-9	≤ 7
**History of Hypertension**	No	Suspect	Yes
**History of Heart Failure**	No	Suspect	Yes
**History of Ischemic Heart Disease**	No	Suspect	Yes
**History of Diabetes**	No	Suspect	Yes
**History of Stroke**	No	Suspect	Yes
**History of Chronic Renal Failure**	No	Suspect	Yes
**History of Chronic Liver Failure**	No	Suspect	Yes
**History of COPD**	No	Suspect	Yes
**History of Cancer**	No	Suspect	Yes
	**TOTAL SCORE**	**/30 =**	
